# Morphological evidence supports splitting of species in the North Atlantic *Sebastes* spp. complex

**DOI:** 10.1371/journal.pone.0316988

**Published:** 2025-02-06

**Authors:** Ingrid Marie Bruvold, Agneta Hansen, Arve Lynghammar, Hannes Höffle, Tanja Hanebrekke, Caroline Aas Tranang, Kjell Nedreaas, Einar Nilssen, Atal Saha, Torild Johansen

**Affiliations:** 1 Institute of Marine Research, Framsenteret, Tromsø, Norway; 2 Faculty of Biosciences, Fisheries and Economics, UiT The Arctic University of Norway, Tromsø, Norway; 3 Institute of Marine Research, Nordnes, Bergen, Norway; 4 Centre for Coastal Research, Department of Natural Sciences, University of Agder, Kristiansand, Norway; Uppsala Universitet, SWEDEN

## Abstract

The redfishes (genus *Sebastes*) are long-lived, commercial species in the North Atlantic. Excessive harvest through decades has led to a decline in the mature population of golden redfish (*Sebastes norvegicus*) in Norwegian waters, which is currently considered severely depleted. Accumulating genetic evidence suggests a more complex structure within this genus in the North Atlantic, which has recently inspired the hypotheses of cryptic species within *S. norvegicus.* Despite apparent genetic divergence between two types, they have yet to be verified morphologically. The morphology of genetically assigned fishes from Norwegian and Greenland waters was investigated using traditional morphometric methods, applying Linear Discriminant Analysis and Random Forest classification procedures to identify and evaluate the performance of descriptive characters. Combined with non-parametric meristic analysis, the results show that features such as beak length and eye diameter provide sufficient discrimination between the proposed cryptic species as well as separating them from the sympatric species *S. mentella* and *S. viviparus*. These findings support the presence of an additional redfish species in the North Atlantic, distinguishable both by morphological and genetic characters. This needs to be taken into consideration in future monitoring and management strategies for North Atlantic redfish.

## Introduction

The genus *Sebastes* (commonly known as redfishes) is a diverse group of mainly demersal fishes represented by approximately 110 species across the Pacific and Atlantic Oceans [1]. Only four species are recognized in the North Atlantic: *Sebastes norvegicus* (Ascanius 1772, formerly *S. marinus*) and *S. mentella* Travin 1951 are distributed across the North Atlantic from the east coast of North America to Novaya Zemlya (Russia), while the sister species *S. fasciatus* Storer 1854 and *S. viviparus* Krøyer 1845 are found in the western and eastern parts of the North Atlantic, respectively [2,3].

The evolutionary origin of the *Sebastes* species has been a topic of great interest among biologists due to the large diversity in morphology and life history traits within the genus [4]. Their diversification from ancestral Pacific populations into the four North Atlantic species is a relatively recent event on the geological time scale. *Sebastes viviparus* branched off from the basal lineage less than a million years ago followed by *S. fasciatus, S. norvegicus,* and *S. mentella* [5]. The phylogenetic relationship between the species has been problematic to establish. They share highly similar external morphology, and the classification of *S. mentella* and *S. norvegicus* as separate species was still a topic of discussion in the 1960’s [6]. The North Atlantic *Sebastes* species are long-lived, slow growing, and mature at a late age [7]. Whilst lifespans of over 60 years have been recorded for *S. norvegicus*, *S. mentella* can live for over 70 years [8,9]. *Sebastes viviparus* is the smallest species of the three [10], reaching a maximum age of 40 years [11].

Correct species identification is vital for sustainable fishery management. Historically, both *S. norvegicus* and *S. mentella* have been commercially important to countries such as Norway, Germany, Russia, Greenland, and Iceland, with the latter performing the majority of redfish harvest in the North Atlantic [12]. Generation times of *Sebastes* species can exceed a decade [13], making them particularly vulnerable to overfishing. Direct fishery for both species in Norwegian waters has been unsustainable, causing population declines over the past decades. Consequently, direct fishery for *S. mentella* and *S. norvegicus* was prohibited in Norwegian waters from 2003 and 2015, respectively [14]. The Northeast Atlantic stock of *S. mentella* has since been rebuilt while *S. norvegicus* is still considered to be severely depleted with a reduced spawning stock and poor recruitment [15]. Since the fishery for *S. mentella* resumed, morphological misclassification between the superficially similar species has become an issue, potentially driving underestimated bycatch rates of *S. norvegicus* [16].

All *Sebastes* species share an ovoviviparous reproductive mode with internal fertilization [13,17] which has been suggested to be a contributing factor for complex mating behaviors. Introgressive hybridization, where genetic flow between hybrid offspring and parent species occurs through backcrossing, has been documented within the North Atlantic *Sebastes* genus [18–21] particularly between *S. mentella* and *S. fasciatus* (see [22]) and between *S. mentella* and *S. viviparus* (see [23]). Interspecific hybridization and its effect on genetic population structure is suspected to be extensive [23] but is largely unexplored in Norwegian waters. This is further complicated by the limited knowledge of *Sebastes* migration patterns and reproductive habits. The complex biology of redfish including reproductive behaviors and specific habitat preference could potentially facilitate the observed rapid genetic divergence in multiple lineages [5,24,25]. This is thought to be of a sympatric or parapatric nature, leaving morphological traits largely retained [5,26,27], which has inspired hypotheses of potentially frequent cryptic speciation.

Among the four established North Atlantic *Sebastes* species, cryptic species and incipient speciation have been suspected within *S. norvegicus* [19,26,28–30] and *S. mentella* [20,31], respectively. Research on the genetic variation within *S. mentella* has previously disclosed three *S. mentella* ecomorphs, delineated by depth and geographic location into ‘deep pelagic’, ‘shallow pelagic’ and ‘slope’ morphs [20,31,32]. These are partially supported by color variation, parasite infection rate [33] and morphometrics [34].Within *S. norvegicus*, a giant form was proposed in the 1960’s [6] and later identified with molecular and morphological studies from East Greenland waters [19,20] and the Reykjanes Ridge [35]. Two additional cryptic species within *S. norvegicus* in Greenland, Iceland, and Faroe Island waters were genetically identified by Schmidt [26], later referred to as *S. norvegicus* type A and type B by Saha et al. [19]. Little is known about the distributions of the two types in Norwegian waters except for the registration of four individuals of type A from tissue sample analysis [19,20].

The genetic divergence between *S. norvegicus* types A and B was found to be similar to the divergence between *S. mentella* and *S. viviparus* with low levels of hybridization [26], exceeding the genetic divergence found between the *S. mentella* ecomorphs [19,20]. The two *S. norvegicus* types A and B have previously not been described morphologically, but Single Nucleotide Polymorphism (SNP) markers have been developed to differentiate between the types based on Saha et al. [19,36] and Johansen et al. [37].

The present study builds upon the hypothesized *S. norvegicus* cryptic species derived from genetic findings [19] as well as observations of morphological variations described in Nedreaas and Nævdal [28,29]. Using a combination of morphometric and meristic methods with molecular support, this study aims to reveal visual diagnostic characters useful for identifying genetically delimited *S. norvegicus* types A and B, as well as separate them from *S. mentella* and *S. viviparus*. The following research questions are in focus: i) Are there morphological differences between the genetically assigned types *S. norvegicus*-A and *S. norvegicus*-B? ii) How are they distributed by area and depth? These questions are vital for facilitating correct identification at sea, providing a basis for conservation purposes.

## Materials and methods

### Sample collection

**Fig 1:**
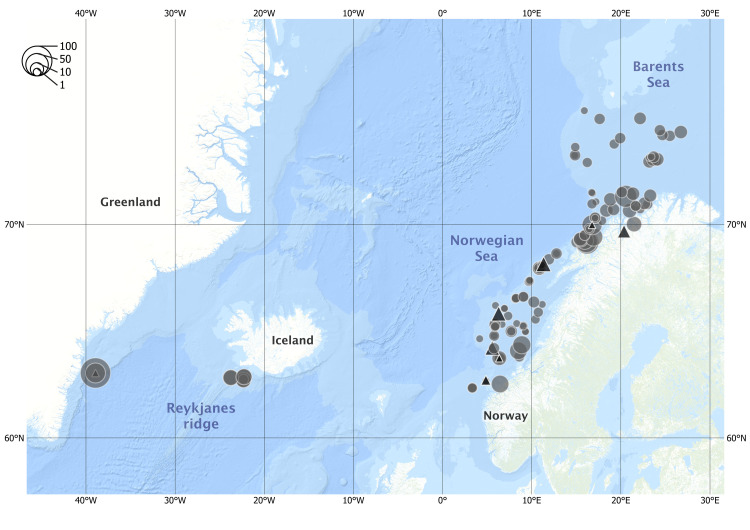
Sampling locations of the 1,170 *Sebastes* spp. collected from three regions: Greenland, Iceland, and Norway. Both whole fish (▲) collected for morphological analysis and archived samples (● ) from the Institute of Marine Research were assigned to species at sea and by genetic analysis in the lab. Point size indicates relative number of individuals (see legend). Map created with ArcGIS® software by Esri. Bathymetry sources: Esri, GEBCO, NOAA, National Geographic, DeLorme, HERE, Geonames.org, and other contributors [38].

In total, we considered 1,170 specimens of *Sebastes* including 992 archived tissue samples from IMR (see below) and 178 whole frozen fish. The samples were collected in spring and fall of 2016 to 2020 from Norway, Greenland and Iceland (Fig 1) by research and commercial vessels using trawls, gillnets, and longlines (Table 1). Most specimens were visually classified to species at sea. The morphological study included *S. norvegicus* and *S. mentella* from Norwegian and Greenland waters, as well as *S. viviparus* and undetermined *Sebastes* from Norwegian waters. Prior to analysis, the specimens were genetically assigned to species or types using three diagnostic Single Nucleotide Polymorphism markers developed at the Institute of Marine Research, Norway [37]. For genetic analysis, fin clips and gill filaments were sampled from all fish and stored in 96% ethanol. Otoliths were collected and stored dry in paper envelopes. The archived samples included fin clips, gill samples, and life history information of *Sebastes* specimens from Norwegian, Greenlandic, and Icelandic waters.

**Table 1:** Overview of the 1,170 sampled *Sebastes* spp. collected for morphological analysis (whole fish) and genetic analysis (archived material) from three regions. All fish were assigned to species at sea and later genetically assigned using tissue samples. When necessary, total length (TL) was estimated from standard length using a conversion formula [39] shown in S1 Table n  =  sample size, BT = bottom trawl, PT = pelagic trawl.

Material	Region	Year	Season	Survey	Classification at sea	n	Depth range (m)			Gear	Age (years)				Length (TL, mm)				Sex		
											Mean	Range			Mean	Range			F	M	NA
Whole fish	Norway	2019	Spring	Research	*S. norvegicus*	10	198	–	198	BT	29	11	–	49	408	360	–	430	1	9	0
				Commercial	Undetermined	18	400	–	400	“	29	19	–	44	397	350	–	430	4	14	0
		2020	Spring	Research	*S. norvegicus*	6	363	–	363	“	33	23	–	45	517	430	–	610	4	2	0
					*S. mentella*	26	458	–	630	“	43	29	–	60	402	350	–	450	14	12	0
					*S. viviparus*	22	390	–	390	“	20	8	–	38	239	200	–	270	14	8	0
			Fall	Research	*S. norvegicus*	29	159	–	214	“	16	9	–	51	367	310	–	440	9	19	1
	Greenland	2020	Spring	Commercial	*S. norvegicus*	38	225	–	225	Longline	24	17	–	36	498	330	–	630	13	18	7
					*S. mentella*	29	225	–	225	“	15	11	–	25	302	260	–	350	0	1	28
Archived	Norway	2016	Spring	Research	*S. norvegicus*	51	296	–	680	BT	30	9	–	50	493	260	–	760	31	20	0
material					*S. mentella*	123	240	–	1018	BT, PT	27	8	–	55	374	240	–	470	79	44	0
		2017	Spring	Commercial	*S. norvegicus*	25	147	–	147	BT	23	16	–	31	438	400	–	480	11	14	0
					*S. norvegicus*	25	70	–	70	Gillnet	23	16	–	33	462	390	–	530	9	15	1
		2018	Spring	Research	*S. norvegicus*	51	241	–	1188	BT	18	9	–	41	455	340	–	760	34	16	1
		2019	Fall	Commercial	*S. norvegicus*	28	NA	–	NA	Gillnet	19	10	–	45	395	330	–	470	17	11	0
					*S. norvegicus*	88	30	–	100	“	14	7	–	47	393	220	–	560	0	0	88
			Fall	Commercial	Undetermined	11	80	–	100	“	15	9	–	30	426	390	–	480	0	0	11
			Spring	Commercial	Undetermined	2	400	–	400	“	32	30	–	34	410	410	–	410	1	0	1
		2020	Spring	Research	*S. norvegicus*	50	303	–	540	BT	29	7	–	63	456	270	–	680	31	19	0
			Spring	Research	*S. viviparus*	8	390	–	390	“	NA	NA	–	NA	NA	NA	–	NA	0	0	8
			Spring	Commercial	*S. norvegicus*	57	100	–	100	Gillnet	13	8	–	30	404	330	–	450	0	0	57
						43	261	–	285	Longline	19	10	–	45	465	330	–	660	15	28	0
			Fall	Research	*S. norvegicus*	215	63	–	379	BT	15	2	–	50	364	110	–	700	96	97	22
					*S. mentella*	4	379	–	379	“	NA	NA	–	NA	453	430	–	490	1	3	0
					*S. viviparus*	3	63	–	207	“	46	46	–	46	297	170	–	490	1	0	2
	Greenland	2020	Spring	Commercial	*S. norvegicus*	66	225	–	225	Longline	27	12	–	45	538	280	–	620	23	23	20
					*S. mentella*	10	225	–	225	“	15	11	–	26	318	260	–	360	1	0	9
	Iceland	2017	Spring	Commercial	*S. norvegicus*	79	247	–	307	“	24	13	–	45	400	250	–	460	31	48	0
						53	247	–	306	“	27	15	–	43	364	260	–	440	21	32	0

### Genetic analysis

DNA was extracted from gill filaments or fin clips using the E-Z 96 Tissue omega DNA Kit (Omega Bio-Teck, Inc.) following the manufacturer’s protocol. The three diagnostic SNPs (Table 2) were developed from ddRAD sequencing of 500 *Sebastes* spp. from the Northeast Atlantic [37], previously identified to species by molecular genetic markers in earlier projects [19,32,35,40]. The SNP markers were selected to diagnostically differentiate between the three common Northeast Atlantic *Sebastes,* as well as identifying the proposed cryptic species *S. norvegicus*-A and B observed by Saha and colleagues [19]*.* The markers SEB29 and SEB39 identified *S. viviparus* and *S. mentella* respectively, while SEB25 separated *S. norvegicus*-A from *S. norvegicus*-B. A set of TaqMan SNP Genotyping assays (Thermo Fisher Scientific, Waltham, USA) was designed for fast identification of species by Johansen et al. [37] and for the present study.

**Table 2:** In total, 1,171 *Sebastes* spp. were analyzed for the three diagnostic SNPs. Heterozygous individuals were identified for SEB29 (n = 1) and SEB25 (n = 33). See text for details.

Assigned species	n	SEB29 *S. viviparus*		SEB39 *S. mentella*		SEB25 *S. norvegicus* A/B	
*S. mentella*	144	C	C	C	C	G	G
*S. norvegicus*-A	140	C	C	T	T	A	A
*S. norvegicus*-B	781	C	C	T	T	G	G
*S. viviparus*	71	T	T	T	T	G	G

The three diagnostic SNP markers were used to genetically assign 1,170 fish into species, independent of morphological identification. An unrooted Neighbour-Joining tree was constructed to visualize the segregation of the *Sebastes* species based on these SNP markers (S1 Fig). The extent of divergence among *Sebastes* spp*.* was quantified by the chord distance (*D*_CE_ [41]). Pair-wise distances were measured to construct an unrooted-phylogram using the Neighbour-Joining (NJ) algorithm [42] available in Populations [43]. We performed 1,000 bootstraps on loci to estimate confidence of nodes in the tree. The phylip format tree generated by Populations was finally viewed in Fig Tree 1.4.2 (http://tree.bio.ed.ac.uk/software/figtree/ Fig Tree v1.4.2).

### Morphological and meristic analysis

**Fig 2:**
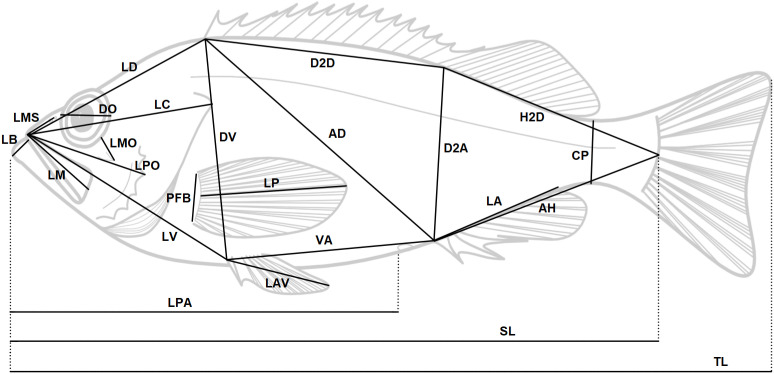
Interlandmark distances measured in traditional morphometrics. Width between opercula (AN, not shown) was measured on the dorsal side. SL = Standard Length, TL = Total Length. See S5 Table for explanation of abbreviations.

**Fig 3:**
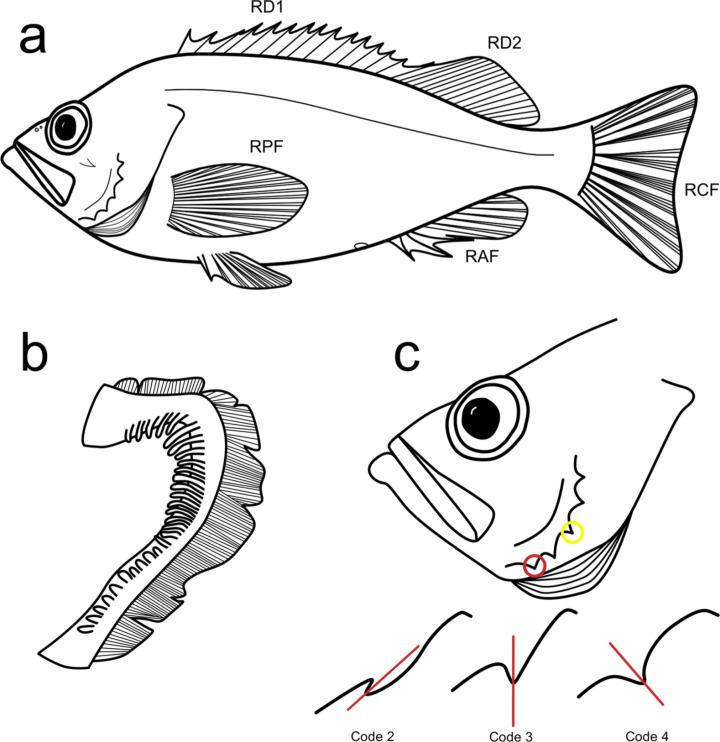
Features included in meristic analysis including a) fin ray counts for the spiny dorsal fin (RD1), soft rays in dorsal fin (RD2), caudal fin (RCF), pectoral fin (RPF), anal fin ray (RAF). Details in b) shows branchial arch with gill rakers (left) and gill filaments (right), and c) illustrates third preopercular spine (PS3, yellow circle) and fifth preopercular spine (PS5, red circle) with recorded angles following the coding system described by Garabana [44].

Morphological analysis was conducted on 178 specimens of *Sebastes* from Greenland and Norway (Table 1) which were defrosted in freshwater overnight prior to examination. All fish were measured to total and standard lengths to the nearest 0.1 mm below on a measuring board. Measurements (n = 23, Fig 2) were taken to the nearest 0.1 mm with digital calipers, and meristic counts (n = 9, Fig 3) were recorded on the left side of the fish when possible, following Garabana [44] and Power and Ni [45]. This included the number of gill rakers (Fig 3B) which were counted on the first gill arch. Angles of the preopercular spines were recorded using a coding system ([44], Fig 3C). All measurements and counts were made by the same person to minimize observational errors. Only a subset of the variables was recorded for the reference samples from Greenland as some variables did not show variations, such as the number of rays in the dorsal, pelvic, and caudal fins.

To identify the optimal combinations of variables for classification under varying species and type compositions, Linear Discriminant Analysis (LDA) was performed for two subsets of data [46]. This is a supervised multivariate approach to classification, accounting for prior genetic assignment. Norwegian specimens of *S. norvegicus*-A, *S. norvegicus*-B, *S. mentella*, and *S. viviparus* were analyzed together, and specimens of *S. norvegicus* types A and B from Norwegian and Greenland waters were compared in a separate analysis. As the assumptions of LDA are often not met for morphometric data [46], a non-parametric Random Forest analysis [47] was performed on the dataset consisting of Norwegian specimens as well as the total dataset including Greenland fish. All analyses were performed using the software R version 4.0.4 [48].

Potential outliers were identified in residuals plotted from regression of all variables against the standard length for each group of genetically assigned fish and removed if deemed illegitimate. When possible, individuals with missing measurements were used in the combined analysis for practical application described below, or otherwise removed from analysis. Measurements were expressed as a fraction of standard length for each individual. Multivariate normality was evaluated by computing Mardia’s skewness and kurtosis (R package ‘*MVN’* [49]) and examining quantile-quantile plots, and the Fligner-Killeen’s test was used to assess homogeneity of variances prior to statistical analyses.

Prior to LDA, dataset dimensionality was reduced through recursive feature elimination. This provides a backward selection of variables based on feature ranking to minimize overfitting and consequentially poor generalization ability of the model [50]. The reduced dataset was partitioned into a training (70%) and testing (30%) subset which were subjected to LDA for classification. These results were compared to the Random Forest procedure through which relative variable importance with permutation was also calculated [51]. Averaged model prediction accuracy of group membership was estimated and compared between LDA and Random Forest using 10-fold cross-validation with 5 repeats, as well as the out-of-bag error measurement calculated by Random Forest.

For individuals with indeterminate genetic results, we predicted their morphological group assignment based on morphometric measurements as part of the classification procedure in LDA.

Meristic counts were analyzed using a Kruskal-Wallis test assessing mean counts across groups [52] and a subsequent post-hoc Dunn test (package ‘*dunn.test’* [53]) identifying between-group differences. The Dunn test was adjusted for comparisons of multiple groups with the Benjamini-Hochberg procedure [54]. Individuals with missing measurements were removed from the dataset but used in the combined analysis for practical application.

To evaluate the performance of morphometric and meristic variables together, a combined analysis was conducted with Random Forest including the most important variables identified in morphological analyses to classify specimens from Norwegian waters (n = 107, S2 Table). This also included individuals removed from the initial morphometric and meristic analyses due to missing measurements.

### Age estimation

The left sagittal otolith was chosen for age determination and prepared using the break-and-burn technique [55]. The otolith was broken in half through the nucleus using a scalpel and held over the flame of an alcohol burner for a few seconds until reaching the desired brown color [56]. The otolith was then mounted in plasticine and the burned surface was coated with mineral oil to enhance the growth zones. Age was determined by two experienced technicians counting the hyaline zones (winter zones) under a stereomicroscope with reflected light.

## Results

### Genetic assignment and age determination

Of the three diagnostic SNPs, SEB29 and SEB39 identified *S. viviparus* and *S. mentella,* respectively. SEB25 separated *S. norvegicus*-A from *S. norvegicus*-B (Table 2). Of the 1,170 genetically assigned specimens, 140 fish were assigned to *S. norvegicus*-A, 781 to *S. norvegicus*-B, 144 to *S. mentella*, and 71 to *S. viviparus* (Table 2, Table 3) while 34 specimens were heterozygous for the SNPs used. Age ranged from two to 63 years for all fish measuring from 110 to 760 mm total length. The morphologically examined fish were determined to be between eight and 60 years old with total length ranging from 200 to 630 mm, excluding any juveniles from the morphological analyses.

**Table 3:** Classification matrix depicting morphological classification at sea and genetic assignment for the 1,170 *Sebastes* spp. from three regions. Numbers in parenthesis show the number of individuals included in morphological analysis.

Region	Genetic assignment	Total (n)	Classification at sea			
			*S. norvegicus*	*S. mentella*	*S. viviparus*	Undetermined
Norway	*S. norvegicus*-A	49	21(1)	12		16(15)
	*S. norvegicus*-B	610	598(43)			12(1)
	Heterozygous SEB25 A/B	23	16	4		3(2)
	*S. mentella*	143	6(1)	137(26)		
	*S. viviparus*	69	37		32(22)	
	Heterozygous SEB39 *S. m*/*S. v*	1			1	
	**Total (n)**	**895 (111)**	**679(45)**	**154(26)**	**33(22)**	**31(18)**
Greenland	*S. norvegicus*-A	36		36(26)		
	*S. norvegicus*-B	104	104(38)			
	Heterozygous SEB25 A/B	2		2(2)		
	*S. mentella*	1		1(1)		
	**Total (n)**	**143 (77)**	**104(38)**	**39(29)**		
Iceland	*S. norvegicus*-A	55	11	44		
	*S. norvegicus*-B	67	66	1		
	Heterozygous SEB25 A/B	8	1	7		
	*S. viviparus*	2	1	1		
	**Total (n)**	**132**	**79**	**53**		

Most specimens classified by visual inspection as *S. norvegicus* at sea were genetically assigned to *S. norvegicus*-B. Genetically identified *S. norvegicus*-A were initially classified as either *S. norvegicus* or *S. mentella* at sea, but more frequently as the latter. The majority of *S. mentella* specimens collected in Greenland waters were genetically assigned to *S. norvegicus*-A, while all *S. mentella* specimens from Icelandic waters were assigned to *S. norvegicus*-B, *S. norvegicus*-A, or resulted heterozygous, with two individuals assigned to *S. viviparus*. None of the specimens from Icelandic waters were genetically assigned to *S. mentella*.

Of all redfish analyzed, only 2.9% (n = 33) of the fish were heterozygous for the SEB25 and could not be assigned either to *S. norvegicus*-A or *S. norvegicus*-B. The heterozygous individuals were observed among the redfish from Norway, Greenland, and Iceland, both in the archived tissue samples (n = 29) and among whole fish for morphometric analysis (n = 4). One redfish from Norwegian waters was heterozygous for the SEB39, the marker separating *S. mentella* and *S. viviparus*. All heterozygote individuals were excluded from the morphological analyses apart from the combined analysis for practical application.

Among the whole fish for morphological analysis from Norwegian waters, 16 were *S. norvegicus*-A, 44 *S. norvegicus*-B, 27 *S. mentella* and 22 *S. viviparus*. This included the undetermined whole fish (see Table 1), of which 16 were assigned to *S. norvegicus*-A, one was assigned to *S. norvegicus*-B. In addition, two fish were heterozygous for the SEB25. Of the whole fish from Greenland, 26 fish were assigned to *S. norvegicus*-A, 38 to *S. norvegicus*-B, and one individual was assigned to *S. mentella*, while two fish were heterozygous for SEB25.

### Morphometric analysis

Out of 178 whole redfish, a total of 99 Norwegian and 30 Greenland specimens were retained in analyses after removing three outlier individuals and eleven specimens with missing measurements. Four individuals were heterozygous for the SEB25, and the remaining 31 individuals were only included in the combined analysis for practical application, as they were missing measurements needed for the full analyses. The morphometric data was found to deviate from multivariate normality and homogeneity of variances. As the main focus of the morphometric analyses was on delimitating the Norwegian specimens, the Greenland specimens were only used as reference sample.

**Fig 4:**
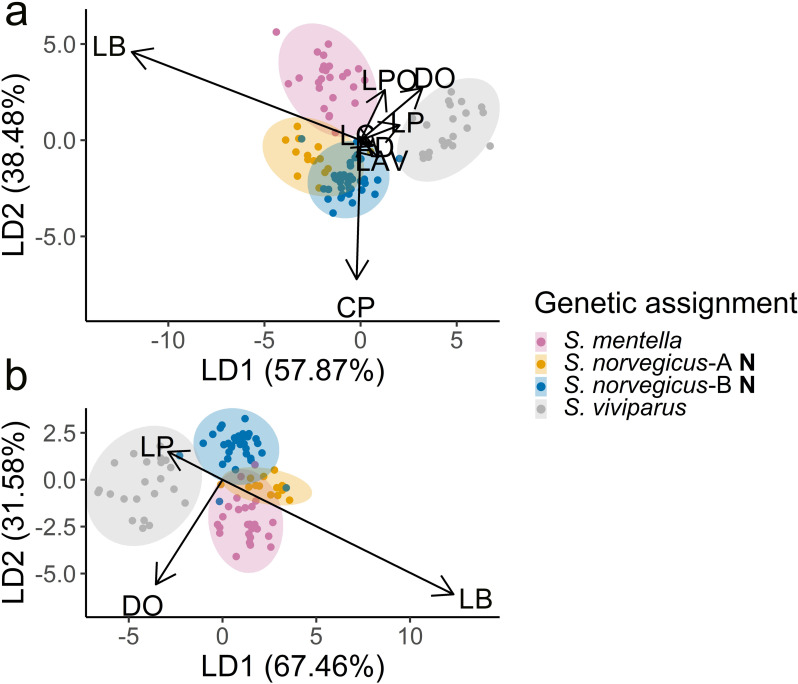
Morphometric ordination of *Sebastes* specimens from Norwegian waters. Panel a) shows biplot from Linear Discriminant Analysis (LDA) based on 8 variables selected by Recursive Feature Elimination, while b) shows biplot for the top three variables eye diameter (DO), beak length (LB), and pectoral fin length (LP). Arrow lengths and directions indicate vector loadings, showing how variables influence the linear discriminants and to what extent. Ellipses show 95% confidence interval for each group.

Among the morphometric variables measured on Norwegian specimens, the recursive feature elimination model selected eight variables for an optimal accuracy. This included eye diameter (DO), beak length (LB), pectoral fin length (LP), distance from snout to preopercular spine (LPO), pelvic fin length (LAV), distance snout to edge of operculum (LC), caudal peduncle height (CP), and distance from the anterior insertion of the dorsal fin to the anterior insertion of the anal fin (AD). The first two linear discriminants in the LDA explained 96.4% of the total variation among specimens from Norwegian waters (Fig 4A) which fully separated *S. norvegicus*-B and *S. norvegicus*-A from *S. mentella* and *S. viviparus* with a cross-validation classification accuracy of 0.95 (Kappa = 0.93). All *S. viviparus* were correctly classified, and classification accuracy was generally high for the genetically assigned *S. mentella*, *S. norvegicus*-A, and *S. norvegicus*-B (S3 Table). By reducing the number of variables for practical application, a dataset containing only the top three characters DO, LB, and LP were provided to the LDA. This model achieved a high degree of separation, with the first and second discriminants explaining 99% of the variation (Fig 4B) with a minor decrease in Kappa to 0.91. Cross-validation classification achieved an accuracy of 0.93 (Kappa = 0.90). When comparing both analyses, LB contributed the most explanatory power to the first discriminant and varied to the greatest degree between groups, as indicated by arrow length. The strongest influence on the second discriminant was CP, while DO and LPO influenced both axes. The group orientations in the biplots suggest that specimens of *S. norvegicus*-A had larger eye diameters as well as longer beaks compared to *S. norvegicus*-B while *S. mentella* and *S. viviparus* had the relatively longest and shortest beak lengths respectively. Specimens of *S. viviparus* had longer pectoral fins than the remaining groups.

#### Norway vs. Greenland.

**Fig 5:**
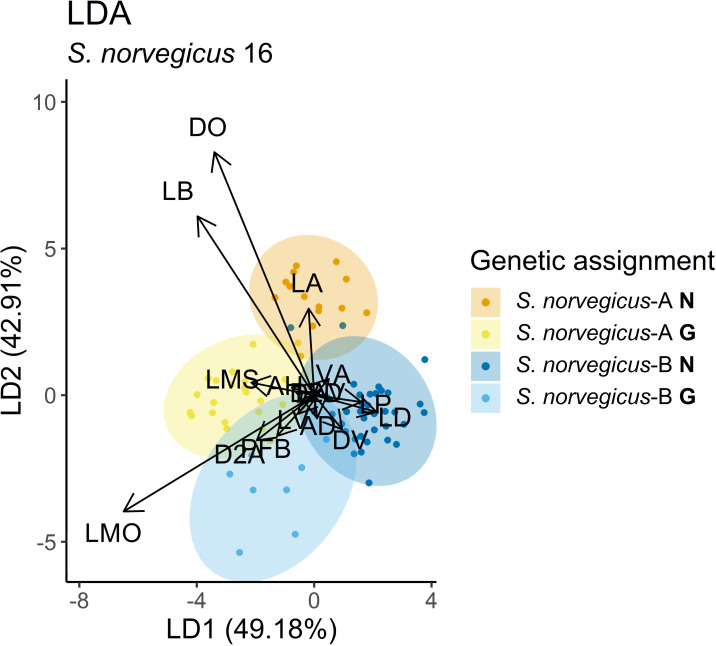
Morphometric ordination based on Linear Discriminant analysis of *S. norvegicus*-A and *S. norvegicus*-B from Norwegian (N) and Greenland (G) waters including 16 variables selected by Recursive Feature Elimination (seeS5 Table for explanation of abbreviations). Arrow lengths and directions indicate vector loadings, showing how variables influence the linear discriminants and to what extent. Ellipses show 95% confidence interval for each group.

The variation between *S. norvegicus*-B and *S. norvegicus*-A was explored further in an analysis of the specimens from Norwegian and Greenland waters. Here, recursive feature elimination selected 16 variables to provide the highest classification accuracy. Although the first two linear discriminants of the LDA accounted for 92% of the variation, overlap between groups in the ordination plot (Fig 5) was reflected in a cross-validation classification accuracy of 0.89 (Kappa = 0.82). The relative ordination of the groups suggested that *S. norvegicus*-A from Norwegian and Greenland waters had relatively longer beaks and larger eye diameter than *S. norvegicus*-B from both Norway and Greenland. The distance between eye and post opercular spine contributed the most to the separation of types A and B within regions. Assigning the heterozygous Norwegian (n = 2) and Greenland (n = 2) whole specimens with the morphological models placed the individuals together with the Norwegian and Greenland *S. norvegicus*-A respectively.

Between-group variable importance was calculated with permutation using Random Forest, which showed that some variables were consistently ranked high across group comparisons. Results were produced for a reduced dataset containing only Norwegian specimens (S2 Fig), as well as the full dataset with Greenland samples (S3 Fig). Between the Norwegian *S. norvegicus*-B and *S. norvegicus*-A, the variables DO, LB, and LPO were ranked highest in variable importance suggesting that these three characters contributed the most to discrimination. The CP was the third most important character for separating both *S. norvegicus*-A and B from *S. mentella* after DO and LB. Compared with *S. viviparus*, DO, LAV, and LP were important characters for identifying *S. norvegicus*-A. In all comparisons involving *S. mentella*, the model frequently ranked CP as an important variable. A consistently high variable importance was attributed to LP and LAV in regarding comparisons with *S. viviparus*. Between the Greenland specimens of *S. norvegicus* types A and B (S2 Fig), the characters DO, LP, and snout to ventral fin length had the highest values for mean decrease in accuracy. Eye diameter was consistently highest ranked in all comparisons including Greenland specimens of *S. norvegicus*-A and *S. norvegicus*-B. Between the two regions Greenland and Norway, DO and LP were highly ranked for *S. norvegicus*-A while DO and LMS were highly ranked for *S. norvegicus*-B.

A 10-fold cross-validation repeated 5 times gave an accuracy of 0.98 (Kappa = 0.98) for the dataset containing Norwegian samples (S4 Table). All specimens were accurately classified apart from one *S. viviparus* classified as *S. norvegicus*-A. When including the Greenland specimens, the accuracy dropped to 0.85 (Kappa = 0.81). Here, 16 out of 93 fish were misclassified largely within *S. norvegicus*-A and *S. norvegicus*-B. The out-of-bag estimates of classification error (overall model error, S4 Table) for the Norwegian dataset and the full dataset including Greenland specimens were 1.41% and 17.2% respectively.

### Meristics

For the meristic counts, 59 Greenland and 87 Norwegian redfish (S2 Table) were analyzed. The Kruskal-Wallis test revealed significant differences between groups for all variables, except for the number of fin rays in the spiny dorsal fin (Table 4). The two species *S. mentella* and *S. viviparus* could be clearly separated by examining the preopercular spines. For *S. mentella* the PS5 was frequently found to point at a forward angle, while all specimens of *S. viviparus* consistently displayed backwards pointing preopercular spines. However, for the Norwegian *S. norvegicus*-A and *S. norvegicus*-B, patterns of preopercular spine angles did not differ as they typically exhibited a down-backwards pointed angle of the third preopercular spine (PS3) and a downwards pointing angle of the fifth preopercular spine (PS5). *Sebastes norvegicus*-A and *S. norvegicus*-B both showed on average more gill rakers (GR) than *S. mentella*, and the number of gill rakers was typically highest in *S. viviparus.*

**Table 4:** Summary of test statistics from Kruskal-Wallis (K-W) test, which tests for differences between groups, and multiple comparison Dunn test on meristic variables. Variables tested include number of pectoral fin rays (RPF), fin rays in the spiny dorsal fin (RD1), soft fin rays in the dorsal fin (RD2), caudal fin rays (RCF), anal fin rays (RAF), no. of gill rakers (GR) and the angles of the 3^rd^ preopercular spine (PS3) and 5^th^ preopercular spine (PS5) for samples from Norway (N) and Greenland (G). For the Dunn test, ranked means between groups are significantly different (*) if they do not share a letter for a given variable.

	RPF	RD1	RD2	RCF	RAF	GR	PS3	PS5
H	88.0	4.8	25.3	16.1	75.5	67.9	89.4	123.5
Df	5	3	3	3	5	5	5	5
p-value K-W	<0.001*	0.184	<0.001*	0.001*	<0.001*	<0.001*	<0.001*	<0.001*
*S. norvegicus-*A **N**	ab		abc	ab	bc	abc	b	b
*S. norvegicus-*A **G**	ab		–	–	b	bc	b	b
*S. norvegicus-*B **N**	b		b	a	b	ab	b	b
*S. norvegicus-*B **G**	c		–	–	a	d	b	b
*S. mentella*	a		a	a	a	a	a	a
*S. viviparus*	d		c	b	c	c	b	c

The Greenland *S. norvegicus* types A and B were found to follow the same pattern of preopercular spine angles as the specimens collected in Norwegian waters (Table 4). However, *S. norvegicus*-B from Greenland waters had fewer gill rakers on average than *S. norvegicus*-B from Norwegian waters, and *S. norvegicus*-A overall. This group was also differentiated by a higher average number of pectoral and anal fin rays. The remaining characters could not be used to separate *S. norvegicus*-A and *S. norvegicus*-B. A one-tailed T-test showed that for the Norwegian and Greenland *S. norvegicus*-B, mean standard length was significantly higher in Greenland specimens (p <  0.05).

#### Combined morphometric and meristic analysis.

The highest ranked characters identified in morphometric and meristic analysis were combined in a Random Forest classification procedure, which included DO, LB, LP, RAF, GR, PS3, and PS5. The dataset including only Norwegian specimens had a classification accuracy of 0.95 (Kappa = 0.93, out-of-bag = 5.8%). Variable importance showed that LB, DO and PS5 were generally of highest importance for all groups. RAF was of lower importance, but more relevant for *S. mentella* and *S. viviparus*. Including Greenland specimens resulted in a lower classification accuracy of 0.81 (Kappa = 0.76, out-of-bag = 24.1%). The majority of misclassifications were among *S. norvegicus*-B specimens, where the model could not perfectly separate specimens of *S. norvegicus* types between regions. Here, LB was less important among *S. norvegicus*-A and *S. norvegicus*-B specimens compared to the Norwegian dataset, while DO remained highly important. GR was particularly important for distinguishing between Norwegian and Greenland *S. norvegicus*-B.

### Geographical and depth distribution

**Fig 6:**
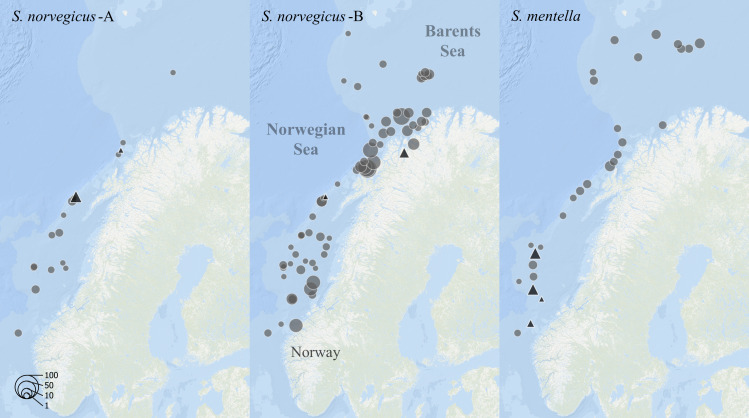
Locations of redfish sampled in Norwegian waters genetically identified as *S. norvegicus*-A (left), *S. norvegicus*-B (middle), and *S. mentella* (right). The point size indicates relative number of individuals (see legend) partitioned between whole fish (▲) for morphometric analysis and archived tissue samples (● ). Map created with ArcGIS® software by Esri. Bathymetry sources: Esri, GEBCO, NOAA, National Geographic, DeLorme, HERE, Geonames.org, and other contributors [38].

Sampled individuals of *S. norvegicus*-A and *S. norvegicus*-B were partially overlapping in the Norwegian and Barents Sea (Fig 6). *Sebastes norvegicus*-A was collected between 62.7°N to 72°N at depths between 266 to 581 meters, while *S. norvegicus*-B was collected even further north (62.7°N to 73.9°N) at 90–706 meters. A mixture of both types as well as *S. mentella* was present in multiple trawl catches. Regarding seasonality, *S. norvegicus*-B were observed in both spring (n = 400) and fall (n = 305). The *S. norvegicus*-A, however, were collected in spring (n = 109) with only a single specimen registered in fall. In spring, several female specimens of both types A and B were found eggs with developing larvae, showing maturation stages equivalent to shortly before, during, or after the larval extrusion period. Mature specimens were also recorded in samples from Greenland and Iceland.

## Discussion

The differences found by both the genetic markers and by morphological examination supports that *S. norvegicus*-A and *S. norvegicus*-B represent separate species. Beak length, eye diameter, caudal peduncle height, and pectoral fin length were consistently emphasized as the most contributing factors to morphometric distinction between the specimens. These characters have historically been among the most important features to differentiate between the North Atlantic *Sebastes* species [44,45].

Previous studies found genetic divergence between *S. norvegicus*-A and *S. norvegicus*-B, similar to that between existing species [36,37]. In the present study we found this reflected in morphometric differentiation, where beak length and eye size contributed the most to morphological separation. Multivariate analyses revealed that the classification accuracy of *S. norvegicus*-A and *S. norvegicus*-B was comparable to the classification accuracy between the recognized species *S. mentella* and *S. viviparus*. The longer beak length in *S. norvegicus*-A compared to *S. norvegicus*-B matches a short description of ten individuals caught in Greenland waters by Schmidt [26]. These specimens were genetically distinct from *S. norvegicus*-B and *S. mentella*, and could potentially represent *S. norvegicus*-A.

High morphological classification success was achieved even based on very few characters (Fig 4B, Kappa = 0.91). Whilst the models separated specimens in Norwegian waters relatively well, slightly higher error rate was observed when the Greenlandic samples were included, which may reflect a more similar morphology between *S. norvegicus*-A and *S. norvegicus*-B in Greenland waters. This could also be influenced by significant differences in length. Despite the closer genetic relationship between the two *S. norvegicus* types, the majority of *S. norvegicus*-A were initially misclassified at sea as *S. mentella*, which can likely be explained by the rather distinct beaks found on both *S. mentella* and *S. norvegicus*-A, and less pronounced in *S. norvegicus*-B.

Meristic characters were successful in separating the *S. norvegicus*-A and *S. norvegicus*-B from *S. mentella* and *S. viviparus*. The preopercular spine angles were found to be similar between *S. norvegicus* types A and B, with the 5^th^ preopercular spine pointing downwards as opposed to the forwards angle often seen in *S. mentella*, while *S. viviparus* consistently displayed backwards pointing spines*.* This reinforces the historical practice of identification based on preopercular spine angles. Although no consistent meristic differences were observed between *S. norvegicus*-A and *S. norvegicus*-B in Norwegian waters, *S. norvegicus*-B from Greenland showed significantly fewer gill rakers. As the number of gill rakers has been found to be negatively correlated with size in redfish [44], this may be caused by unequal length distributions between the Greenland and Norwegian specimens examined with mean lengths of 502 and 399 mm respectively (p <  0.05) which is not accounted for in the meristic analysis. However, fewer gill rakers have also been observed for the giant type of *S. norvegicus* caught on the Reykjanes ridge close to Iceland [6,40]. Further diet analyses could be conducted to determine whether differences in gill raker numbers are linked to potential adaptive behaviors or diet between *S. norvegicus*-A and B, as well as the the giant type.

Due to intraspecific morphological variation, a combination of morphometric characters is required for accurate classification at sea – in line with what Power and Ni [45] suggested to separate *S. mentella* and *S. norvegicus*. For morphologically separating *S. norvegicus*-A and *S. norvegicus*-B, we recommend using a combination of eye diameter, beak length, and caudal peduncle height. Larger eye diameters and longer beaks are more pronounced among the Norwegian specimens of *S. norvegicus*-A, whereas *S. norvegicus*-A from Greenland have slightly smaller eyes and beaks in comparison. The two *S. norvegicus* types can also be distinguished from *S. mentella* using the same morphometric characters, where the eye and beak are largest in *S. mentella* while caudal peduncle height is comparatively the narrowest. As color has previously been identified as an important feature distinguishing *Sebastes* spp. [33], fresh specimens of *S. norvegicus* types and *S. mentella* should be further examined to identify possible color variations. Based on previous literature, it is likely that both *S. norvegicus*-A and *S. norvegicus*-B have orange, golden red coloration distinct from the pink color of *S. mentella* [28,29]. Regardless of color, our classification results show that the *S. norvegicus*-A and *S. norvegicus*-B can be separated based on morphometric characters only.

Geographical overlap in the distribution of *S. norvegicus*-A and *S. norvegicus*-B in Norwegian waters along the continental shelf break and in the Barents Sea as well as the presence of both types in single hauls could suggest that they exist in sympatry in line with the findings of Schmidt [26] and Saha et al. [19]. While *S. norvegicus*-B has been observed across the North Atlantic, *S. norvegicus*-A has previously been found in the area around Faroe Islands, Greenland, and Iceland [26] and until the present work, only four *S. norvegicus*-A have been detected in Norwegian waters [19]. However, it is very likely that it was *S. norvegicus*-A that Nedreaas and Nævdal [28,29] also noted on the Norwegian shelf in the late 1980s. Among the samples in our study, *S. norvegicus*-B was considerably more abundant than *S. norvegicus*-A. The *S. norvegicus* type A described by Saha et al. [19] is equivalent to the *S. norvegicus*-A presented in this study [37], thereby expanding its known range to include the Norwegian shelf and the Barents Sea.

While depth and substrate preferences have contributed to establishing barriers for several cryptic North Pacific *Sebastes* spp. [5], partially overlapping distribution of *S. norvegicus*-A and *S. norvegicus*-B indicates that this case of divergence relies on additional mechanisms for reproductive isolation. The life history of *Sebastes* provides great potential for speciation events either partially in sympatry, or for periods of reproductive isolation allowing for allopatric speciation where they remain geographically separated [5]. Observations of females carrying larvae of both *S. norvegicus*-A and *S. norvegicus*-B coincide in time and space with the described larval release of *Sebastes* along the coast of Norway [9,13]. It remains unclear whether the historical registrations of *S. norvegicus* larval release can be attributed to *S. norvegicus*-A, *S. norvegicus*-B, *S. mentella*, or a mixture of all three depending on the accuracy of morphological identification. Larval release could contribute to reproductive barriers, as shifts in timing or area can prevent the mixing of larvae between populations through altered dispersal [57]. Redfish eggs are not fertilized immediately after copulation but delayed due to storage of sperm [7]. Therefore, potential observations of reproductive overlap do not necessarily provide sufficient information about the timing of copulation or larval extrusion to make inferences about reproductive habits or gene flow between *S. norvegicus*-A and *S. norvegicus*-B. Furthermore, most *S. norvegicus*-A specimens were collected in spring, with only one specimen collected in autumn located in the northern part of the Norwegian shelf. Previous studies have suggested that seasonal migrations can cause different populations to aggregate in certain areas [58]. It is unclear whether our sampled specimens for morphological analysis and the archived material represent a single or multiple populations of *S. norvegicus-*A as we only used the three diagnostic SNP markers to ID them to species or types. Considering that historical records of all aspects of biology and ecology of *S. norvegicus* have been made based on the assumption that only a single *S. norvegicus* species is represented, an effort should be made to delimit and describe the biology and ecology of *S. norvegicus-A* and *S. norvegicus-B* separately in more detail.

The identification of *Sebastes* species by external morphology in the field is challenging [44]. Cryptic speciation and development of reproductive isolation could be masked by a less rapid evolution in external morphology facilitated by homogenous environmental conditions that favor the retention of the current biological expression in redfish [59]. It can be challenging to set the criteria for recognizing and establishing boundaries between species, but instances of cryptic speciation have commonly been uncovered among Pacific *Sebastes* species through morphological and genetic studies [60–64]. The traditional definition of a biological species is based on the reproductive isolation of a group of organisms, with hybrid offspring incapable of reproducing [65]. However, this narrow definition does not necessarily cover the range of genetic, ecological, behavioral, physiological, morphological, and evolutionary traits of separately evolving metapopulations [66]. The rapid evolutionary divergence observed and assumed gene flow between North Atlantic *Sebastes* spp. support a theory of recent speciation [5], where evidence of hybridization and introgression show that reproductive isolation between the established species is incomplete [26]. A shift in morphology while occupying the same range could be driven by ecological divergence, indicated by a potential association between the traits found to be the most different between the types A and B (such as eye size, caudal peduncle height, and beak length) with locomotion and dietary differences [67].

Genetic assignment of the fish showed that a small fraction of specimens from all three areas were heterozygous for the SEB25 separating *S. norvegicus*-A and *S. norvegicus*-B, and one fish displayed heterozygosity for the SEB39 marker identifying *S. viviparus* and *S. mentella*. The appearance of heterozygous individuals for otherwise diagnostic markers could indicate that there are genetic variations within the groups or possible hybridization between them. However, the morphological assignment of the four heterozygotes in SEB25 in the morphological data suggested that the individuals belonged to *S. norvegicus*-A. The heterozygous individuals could also indicate ongoing gene flow between *S. norvegicus*-A and *S. norvegicus*-B. As genetic assignment was inconclusive for 34 (3%) of the *S. norvegicus* individuals, additional markers may be required to capture the full genetic variation within and between the types.

## Conclusions

Here, we reveal that both *S. norvegicus*-A and *S. norvegicus*-B are identifiable by morphological characters in Norwegian, Greenland, and Icelandic waters. In particular, eye diameter, beak length, and caudal peduncle height are important characters for delimitation of adult *S. norvegicus* types as well as separation from *S. mentella* and *S. viviparus*, for which color and meristic characters can also be useful. These traits linked to *S. norvegicus*-A and *S. norvegicus*-B coincide with previous observations of morphological variation, providing an explanation to specimens of *S. norvegicus* resembling *S. mentella*. As morphological elements are partially overlapping, characters such as fresh color variations should be further investigated to assist in accurate visual classification on the individual level.

Geographical and depth distribution of samples show that *S. norvegicus*-A and *S. norvegicus*-B are overlapping both in catch locations and in depth, hence strengthening the notion of sympatric speciation. We should explore the potential for practical application of the characters as a basis for identification in the field both for adult specimens as well as for juvenile fish study for potential ontogenetic shifts. In addition, the implications of additional *Sebastes* species should be considered in future revisions of conservation practices to ensure proper management. This is especially relevant for the long-lived *Sebastes* species, which are particularly susceptible to overfishing.

## Supporting information

S1 Table Metrics used for conversion from standard length to total length used with the formula [64]. (DOCX)

S2 Table Data on length, age and sex of Sebastes spp. included in morphometric analysis divided by collection area. NA=Not available. Asterisk indicates specimens not included in training models for morphometric analysis but classified as part of the testing dataset.
(DOCX)

S3 Table Confusion matrix produced by cross-validated linear discriminant analysis models showing percentage prediction of specimens to group based on morphometric measurements against a priori genetic assignment. Darker grey values indicate higher percentage of predicted specimens.
(DOCX)

S4 Table Confusion matrix produced by random forest classifier based on cross-validation showing number of specimens predicted to group based on morphometric measurements against a priori genetic assignment. Darker grey values indicate higher number of predicted specimens. OOB =  Out-of-bag metric produced by Random Forest describing overall error rate.
(DOCX)

S5 Table List of abbreviations and explanations for morphometric variables measured. (DOCX)

S1 Fig Unrooted Neighbour-Joining tree visualizing the Sebastes species based on three Single Nucleotide Polymorphism markers. (DOCX)

S2 Fig Pairwise plots showing relative morphometric variable importance between Norwegian Sebastes spp. ranked by random forest permutation. (DOCX)

S3 Fig Pairwise plots showing relative morphometric variable importance between Norwegian Sebastes spp. ranked by random forest permutation. (DOCX)
