# Supplementary material for: Morphological evidence supports splitting of species in the North Atlantic Sebastes spp. complex
Source: PLoS One. 2025 Feb 6;20(2):e0316988. doi: 10.1371/journal.pone.0316988 (PMC11801727; doi:10.1371/journal.pone.0316988)

Supplementary information

Table S1. Metrics used for conversion from standard length to total length used with the formula $SL=a+b*TL$ [64].


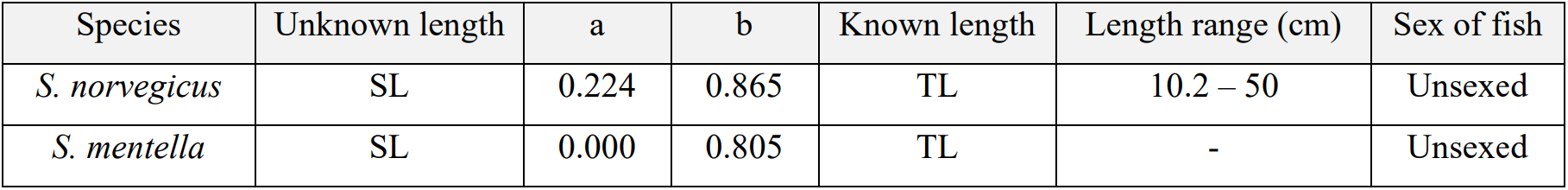

Supplement: S1 Table — (DOCX) [file pone.0316988.s001.docx]
