# Supplementary material for: Morphological evidence supports splitting of species in the North Atlantic Sebastes spp. complex
Source: PLoS One. 2025 Feb 6;20(2):e0316988. doi: 10.1371/journal.pone.0316988 (PMC11801727; doi:10.1371/journal.pone.0316988)
Supplement: S2 Table — NA=Not available. Asterisk indicates specimens not included in training models for morphometric analysis but classified as part of the testing dataset. (DOCX) [file pone.0316988.s002.docx]

Supplementary information

Table S2. Data on length, age and sex of Sebastes spp. included in morphometric analysis divided by collection area. NA=Not available. Asterisk indicates specimens not included in training models for morphometric analysis but classified as part of the testing dataset.
