# Supplementary material for: Morphological evidence supports splitting of species in the North Atlantic Sebastes spp. complex
Source: PLoS One. 2025 Feb 6;20(2):e0316988. doi: 10.1371/journal.pone.0316988 (PMC11801727; doi:10.1371/journal.pone.0316988)
Supplement: S3 Table — Darker grey values indicate higher percentage of predicted specimens. (DOCX) [file pone.0316988.s003.docx]

Supplementary information

Table S3. Confusion matrix produced by cross-validated Linear Discriminant Analysis models showing percentage prediction of specimens to group based on morphometric measurements against a priori genetic assignment. Darker grey values indicate higher percentage of predicted specimens.
