# Supplementary material for: Morphological evidence supports splitting of species in the North Atlantic Sebastes spp. complex
Source: PLoS One. 2025 Feb 6;20(2):e0316988. doi: 10.1371/journal.pone.0316988 (PMC11801727; doi:10.1371/journal.pone.0316988)
Supplement: S4 Table — Darker grey values indicate higher number of predicted specimens. OOB = Out-of-bag metric produced by Random Forest describing overall error rate. (DOCX) [file pone.0316988.s004.docx]

Supplementary information

Table S4. Confusion matrix produced by Random Forest classifier based on cross-validation showing number of specimens predicted to group based on morphometric measurements against a priori genetic assignment. Darker grey values indicate higher number of predicted specimens. OOB = Out-of-bag metric produced by Random Forest describing overall error rate.
