# Supplementary material for: Morphological evidence supports splitting of species in the North Atlantic Sebastes spp. complex
Source: PLoS One. 2025 Feb 6;20(2):e0316988. doi: 10.1371/journal.pone.0316988 (PMC11801727; doi:10.1371/journal.pone.0316988)
Supplement: S5 Table — (DOCX) [file pone.0316988.s005.docx]

Supplementary information

Table S5. List of abbreviations and explanations for morphometric variables measured.
