# Supplementary material for: Morphological evidence supports splitting of species in the North Atlantic Sebastes spp. complex
Source: PLoS One. 2025 Feb 6;20(2):e0316988. doi: 10.1371/journal.pone.0316988 (PMC11801727; doi:10.1371/journal.pone.0316988)
Supplement: S2 Fig — (DOCX) [file pone.0316988.s007.docx]

Supplementary information


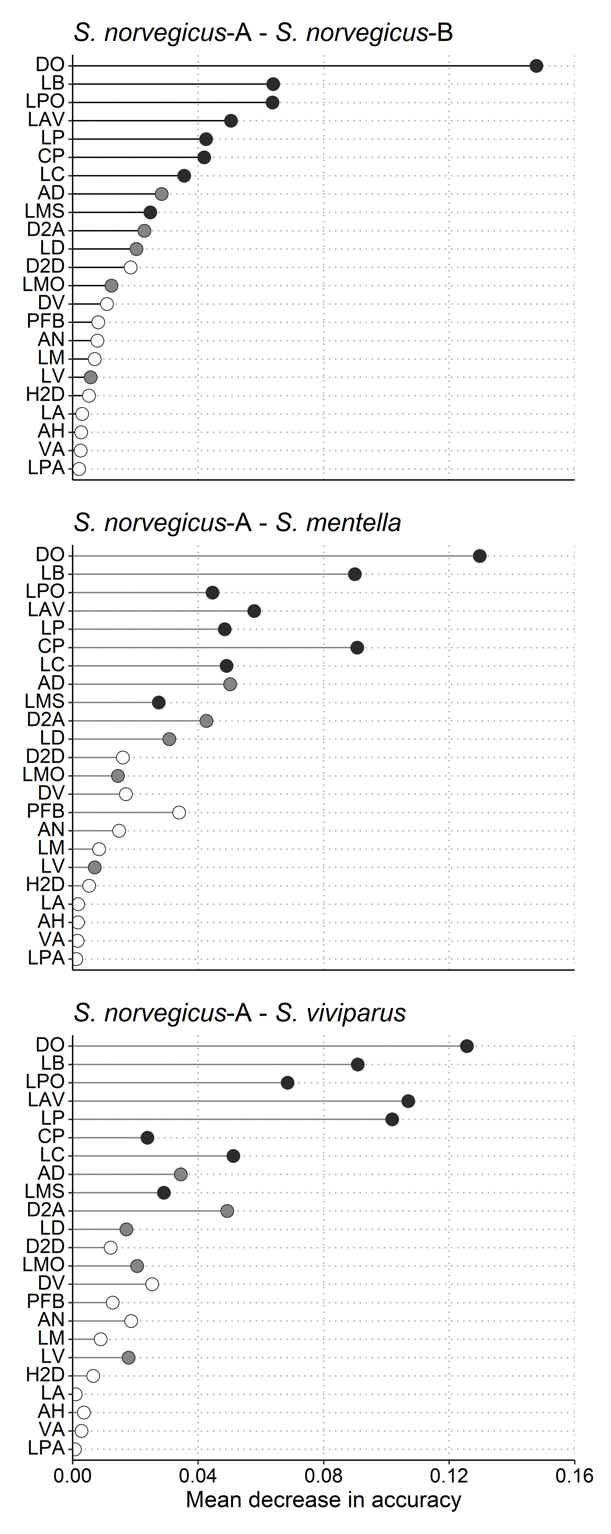

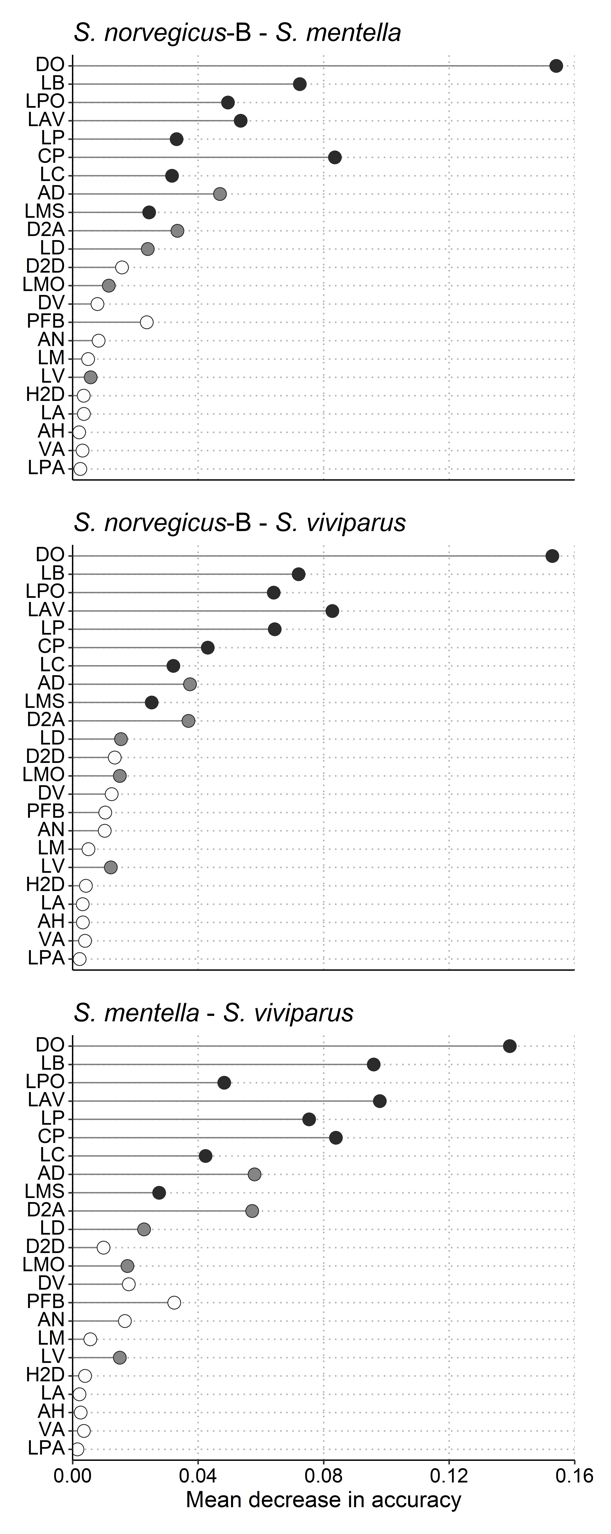


Figure S2. Pairwise plots showing relative morphometric variable importance between Norwegian Sebastes spp. ranked by Random Forest permutation. Black points show variables selected by Recursive Feature Elimination (RFE) as most important for the subset with Norwegian specimens, grey points show additional variables only selected by RFE as important for the full dataset, and white points were not selected for any models. Mean decrease in accuracy describes the loss of classification accuracy with the removal of a given variable. Morphometric variables: Eye diameter (DO), snout to preopercular spine (LPO), beak length (LB), pectoral fin length (LP), pelvic fin length (LAV), snout to edge of operculum (LC), caudal peduncle height (CP), snout to upper nostril (LMS), anterior insertion of 1^st^ spiny ray in dorsal fin to anterior insertion of anal fin (AD), insertion of 1^st^ soft ray in dorsal fin to anal fin (D2A), anterior insertion of 1^st^ spiny ray in dorsal fin to pelvic fin (DV), eye to post-ocular spine (LMO), snout to anterior insertion of 1^st^ spiny ray in dorsal fin (LD), length of spiny rayed dorsal fin base (D2D), maxilla length (LM), neck width (AN), snout to pelvic fin (LV), pectoral fin base width (PFB), anal fin base length (LA), insertion of 1^st^ soft ray in dorsal fin to hypural (H2D), anal fin to hypural (AH), pre-anal length (LPA), pelvic fin to anal fin (VA).
